# Butterfly Patients in Iran Waiting to Specific Attention from Authorities

**Published:** 2017-07

**Authors:** Mohammad Mahdi PARVIZI, Nasrin SAKI, Zahra PARVIZI

**Affiliations:** 1. Molecular Dermatology Research Center, Shiraz University of Medical Sciences, Shiraz, Iran; 2. Health Policy Research Center, Shiraz University of Medical Sciences, Shiraz, Iran; 3. Dept. of Dermatology, School of Medicine, Shiraz University of Medical Sciences, Shiraz, Iran

## Dear Editor-in-Chief

Epidermolysis bullosa (EB), as butterfly disease is a hereditary genetic disease characterized by different degrees of skin and mucosal fragility (1). The pathogenesis of this rare disease involves mutation in skin structural proteins resulting in four major types of EB, including EB simplex, junctional EB, dystrophic EB and Kindler’s syndrome based on ultrastructural mutation level in skin and mucosa (2). The prevalence of EB in United State is eight in one million people and the incidence was 19 in one million alive neonates during 1986 to 1990 (3). The incidence of EB was estimated in America, Australia, and United Kingdom to be 3.5, 10 and 32 per million, respectively (4).

These patients suffers from many physical complications including infection, upper and lower extremities deformities, severe itching, widespread skin ulcerations that make them susceptible to skin cancer, severe chronic constipation, dysphasia and odynophagia due to narrowing of the esophagus. Dental problems, oral mucosal involvements, urinary tract dysfunction and kidney fibrosis are other complications (5).

In several countries, specific foundations are dedicated to these patients such as Dystrophic Epidermolysis Bullosa Research Association of America (DEBRA) in America and Australasian EB Registry in Australia (6). However, there was no registration system for EB patients in Iran until Sep 2015 to detect EB patients. At that time, Iran EB Home was established in Tehran, Iran with the support of the Ministry of Health, Islamic Revolution Mostazafan Foundation and Charity Foundation for Special Diseases. However, the activities of this foundation are limited due to financial issues. Therefore, detailed information is not available about incidence and prevalence of EB in Iranian population.

Iranian patients with EB and their parents are prone to several psychological problems such as anxiety, stress, and depression due to the nature of the disease. In addition, patient’s isolation from the community and social rejection of EB patients due to their physical conditions are two of their major problems. School managers may refuse to register students suffering EB so that they are often deprived of their social rights. Many patients may get worse due to lack of knowledge about their deprivation and disabilities, or inaccessibility of adequate and necessary equipment. These patients can be supported by virtual education. Cultural and educational programs for these patients are major parts of their essential needs to prevent or postpone many of their psychological and physical complications. Community awareness about this disease will increase their social acceptance and social support that can be a balm to their pains and problems.

Although today, some good activities are happening in Iran with the help of Ministry of Health and Medical Education to register and support EB patients. We request the government to define EB as a “Special Disease”, like other diseases such as thalassemia, hemophilia, and dialysis-dependent patients, to facilitate their process of treatment and to define support services for EB patients and their families with the goal of improving their quality of life. EB patients need social and government supports to survive.
